# ASO Author Reflections: Added Value of Pretreatment CT Radiomics for Accurate Prediction of Liver Hypertrophy Following Portal Vein Embolization

**DOI:** 10.1245/s10434-024-16654-2

**Published:** 2024-12-07

**Authors:** Qiang Wang, Torkel B. Brismar, Dennis Björk, Erik Baubeta, Gert Lindell, Bergthor Björnsson, Ernesto Sparrelid

**Affiliations:** 1 Department of Clinical Science, Intervention and Technology (CLINTEC), Division of Medical Imaging and Technology, Karolinska Institutet, Stockholm, Sweden; 2 Department of Radiology, Karolinska University Hospital Huddinge, Stockholm, Sweden; 3 Department of Surgery, Linköping University Hospital, Linköping, Sweden; 4 Department of Imaging and Functional Medicine, Skåne University Hospital, Lund, Sweden; 5 Diagnostic Radiology, Department of Translational Medicine, Lund University, Malmö, Sweden; 6 Department of Surgery, Skåne University Hospital Comprehensive Cancer Center, Clinical Sciences Lund, Faculty of Medicine, Lund University, Lund, Sweden; 7 Division of Surgery and Oncology, Department of Clinical Science, Intervention and Technology, Karolinska Institutet, Karolinska University Hospital, Stockholm, Sweden

## Past

Portal vein embolization (PVE) has been a standard procedure used preoperatively to induce growth of the future liver remnant (FLR). The goal with PVE is to ensure that a sufficient liver volume remains after resection to maintain function in patients with liver malignancies intended for extensive resections. However, predicting which patients will achieve an adequate FLR growth after PVE remains challenging. Current prediction methods are based on clinical factors such as baseline liver function, underlying liver conditions (for instance cirrhosis), and other patient factors. Despite their utility, these predictors often lack the precision required for effective individualized treatment planning. Previous studies have highlighted the need for prediction models that are accurate and accessible, without requiring invasive procedures or costly imaging alternatives.^1^ Our study aimed to bridge this gap by building a model that integrates clinical data with radiomic features derived from routine CT images to accurately predict insufficient liver hypertrophy following PVE.^2^

## Present

In our research, we developed and validated a model that integrates three independent clinical factors (standardized FLR before PVE, ALT levels, and the type of embolization material) with a radiomic signature derived from pre-PVE CT scans. The radiomic signature, which is assumed to capture complex textural and structural patterns within the liver tissue,^3^ provided additional dimensions of data about liver growth potential. Our findings indicate that the combined model achieved higher predictive accuracy than the clinical model alone, with an increase in the area under the curve (AUC) values across derivation and external test cohorts. Although this increase did not reach statistical significance, the improved performance across cohorts is a promising step toward integrating radiomics in routine preoperative assessment.

The clinical significance of our model lies in its potential to serve as a noninvasive, widely accessible tool to help surgeons stratify patients by the risk of insufficient hypertrophy. By extracting data from routine CT scans, this approach avoids the need for additional imaging or invasive tests, making it a practical option for widespread clinical use. For patients deemed at higher risk of inadequate FLR hypertrophy, alternative treatment strategies, closer monitoring, rescue procedures, or even reconsideration of surgical plans could be enabled by this predictive method. Additionally, our model contributes to ongoing efforts to incorporate artificial intelligence (AI) and machine learning in clinical decision-making, demonstrating how radiomics can complement traditional data in the quest for more personalized and precise patient care.^4^

## Future

Future research should focus on expanding and refining this model to increase its predictive accuracy and generalizability. Larger, multicenter studies with diverse patient populations would be valuable to validate our findings and ensure the model’s robustness across different clinical settings. Furthermore, with novel techniques, such as liver venous deprivation (i.e., PVE + hepatic vein embolization) emerging for liver hypertrophy induction in recent years,^5^ the efficacy of our model requires further investigation in these new scenarios. In addition, understanding the biological and genetic foundations of radiomic features remains an open area of exploration. By conducting radiogenomic studies, we may uncover specific genetic or molecular drivers linked to liver regeneration capacity, which could inform future iterations of our model and enhance its predictive capabilities.

In conclusion, our study highlights the value of radiomics in supplement to traditional clinical factors for accurate prediction of liver hypertrophy after PVE. As radiomics and AI continue to evolve, we anticipate such tools to be routinely incorporated into preoperative workflows capable of supporting truly individualized treatment plans in the future.
